# Good and Bad Medicines

**Published:** 1873-03

**Authors:** 


### GOOD AND BAD MEDICINES.

If all medicine is poisonous, never doing any good, but always harm, the more it is adulterated with innocuous articles as flour or magnesia, the better; but if medicines are valuable as a means of health, human life often hangs on their efficiency, hence immeasurable responsibility attaches itself to chemical manufacturers. The American Pharmaceutical Association reports forty different medicines very largely adulterated; and as medicines are sold at a profit of from one to five hundred per cent. the greed of those who make and adulterate them ought to subject them to severe punishment; among those largely diluted are alum, arsenic, bismuth, chloral hydrate, chloroform, cream of tartar, Epsom salts, morphia, quinine, opium, all spices largely. In this way druggists get five dollars a pound for flour and quite as much for water. It is almost as impossible to get morphia or quinine without flour as it is to get now a pure article of Maderia or Port wine. Drunkards are not the only persons killed off by spurious drinks; infants and little children who are fed to death on swilled milk are not the only sufferers from human cupidity.

But perhaps the most remorseless deceptions are practiced in white lead or paint, and out of which most other paints are made. Persons who must have a pure article understand pretty well that there is not an ounce of pure white lead of American manufacture to be bought in the United States. The best houses adulterate here from ten to twenty-five per cent. Scores of thousands of pounds of white lead are sold every year, containing less than twenty-five per cent. of the pure article, the remainder is barytes, a species of earth dug in New York State in large quantities, and can be sold at a profit at half a cent a pound. Counting white lead at ten cents a pound, the measure of profit is immense. The adulteration is so common and so barefaced that manufacturers do not pretend to deny it, but claim that a certain per centage "improves" the white lead. When white paint out of doors turns dark in a short time, it is proof that it contains barytes to a large extent. How it is that white paint is improved by adding a material to it which turns it black has not been explained. The truth is, there is a great opening for honest dealers in all departments of trade. All over New York, and doubtless elsewhere, the men who are known to sell the best article and to do the best work invariably, will always have as much business as they can do at fat prices, from those who always have the money to pay.

There are not three butchers in the city of New York, who could be relied upon to serve the best porter-house steak every day. The only possible way of getting a best cut is to point out with your finger the part you want, see it cut off, deposited in your basket and sent home by your own servant. No family provider would think it possible to get a green-grocer to send a best melon every day for table use, even if the very highest market price is promptly paid on delivery. His calculation would be that it was so near the mark that you would not take the trouble to send it back or that it would not be scrutinized closely; and thus it is that a new departure is necessary to be taken in the education of children; more pains should be taken and more time consumed in inculcating moral precepts, and less in mere learning.

